# Supplementary material for: Enrichment of Aldolase C Correlates with Low Non-Mutated IDH1 Expression and Predicts a Favorable Prognosis in Glioblastomas
Source: Cancers (Basel). 2019 Aug 23;11(9):1238. doi: 10.3390/cancers11091238 (PMC6770576; doi:10.3390/cancers11091238)
Supplement: Supplementary file 1 [file cancers-11-01238-s001.pdf]

cancers

MDPI

Supplementary Materials: Enrichment of Aldolase C Correlates with Low Non-Mutated IDH1 Expression and Predicts a Favorable Prognosis in Glioblastomas

Yu-Chan Chang [1,†], Hsing-Fang Tsai [1,2,†], Shang-Pen Huang [1,3], Chi-Long Chen [4,5], Michael Hsiao [1,6,*] and Wen-Chiuan Tsai [2,*]

Table S1. Clinical relevance of ALDOC expression in glioblastoma (GBM) patients.

		ALDOC expression, *n* (%)		
**Characteristics**	***n***	**Low (*n* =47)**	**High (*n* =42)**	***p*-Value**
Histological type				
Anaplastic Astrocytoma	10	8(57.7)	2(42.3)	
Anaplastic				0.257
Oligodendroglioma	6	3(50.0)	3(50.0)	
Classical glioblastoma	8	3(37.5)	5(62.5)	
Diffuse astrocytoma	14	6(42.9)	8(57.1)	
Diffuse midline glioma	14	6(42.9)	8(57.1)	
Mesenchymal glioblatoma	11	7(63.6)	4(36.4)	
Neural glioblastoma	13	9(69.2)	4(30.8)	
Oligodendroglioma	4	1(25.0)	3(75.0)	
Pilocytic astrocytoma	1	0(0.0)	1(100.0)	
Proneural glioblastoma	8	4(50.0)	4(50.0)	
IDH1 status				
Wild-type	58	36(62.1)	22(37.9)	0.043
Mutant	17	5(29.4)	12(70.6)	
K27M	14	6(42.9)	8(57.1)	
Grade[#]				
I+ II	19	7(36.8)	12(63.2)	0.116
III+ IV	70	40(57.1)	30(42.9)	

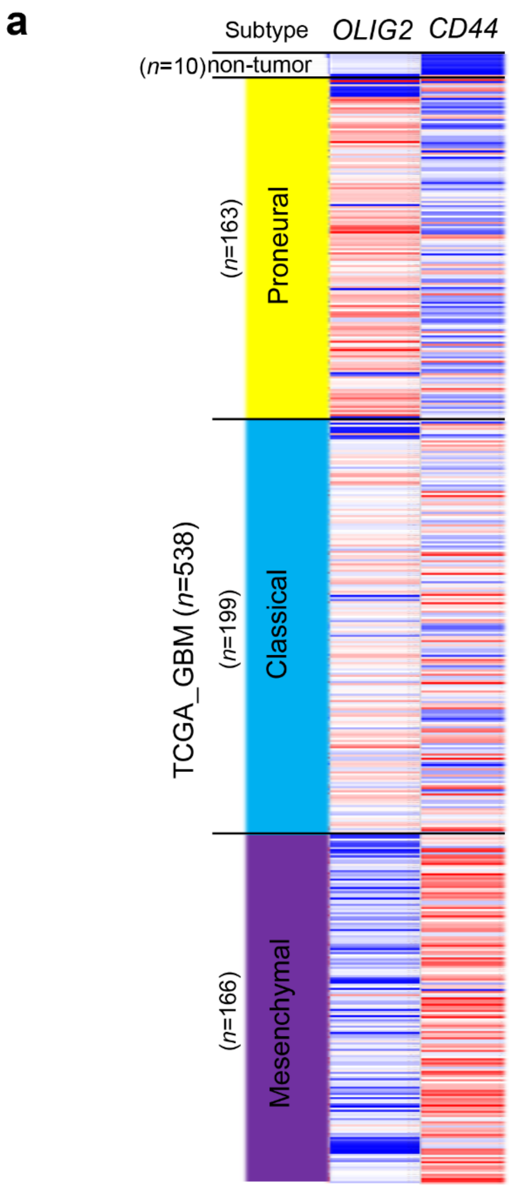

Figure S1. *OLIG2* and *CD44* mRNA expression in various subtypes of GBMs. A heat map showing the endogenous mRNA expression level of *CD44* (marker of mesenchymal type), and the non-mesenchymal marker *OLIG2* in The Cancer Genomics Atlas (TCGA)_Glioblastoma patients by molecular pathology classified (n = 538).

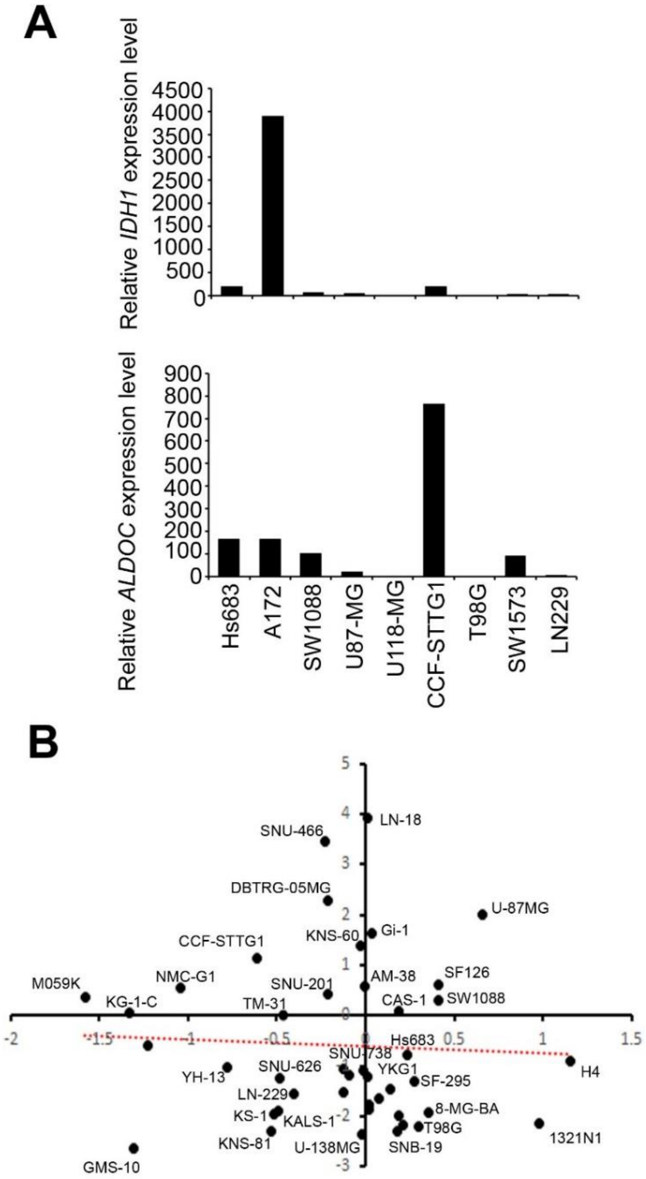

Figure S2. (**A**) The expression of *IDH1* and *ALDOC* in the glioma cancer cell lines panel. (**B**) The correlation between the ALDOC mRNA level and the expression level of *IDH1* in glioblastoma cell patterns from the Cancer Cell Line Encyclopedia (CCLE) cohort. The significance of the differences in B was analyzed using a Student's *t*-test. All cut-off values were set before analysis, and all tests were two-tailed.

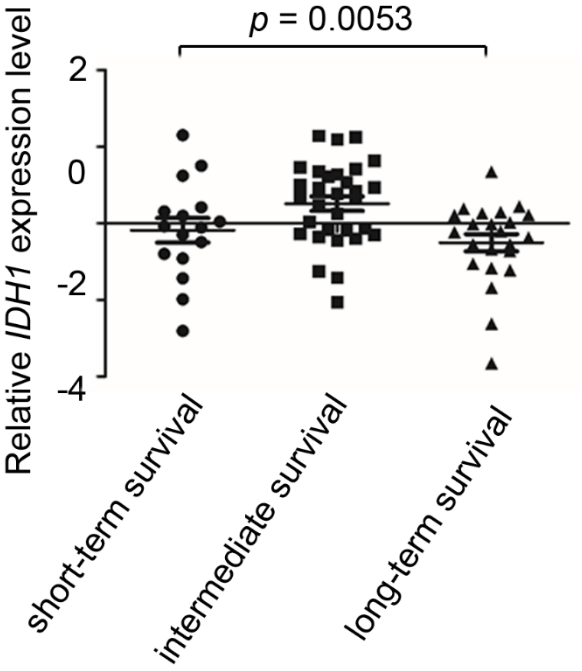

Figure S3. Boxplot showed the distribution of *ALDOC* expression in clinical patients according to the *IDH1* mutant event in the GSE36245 cohort (p = 0.0384).

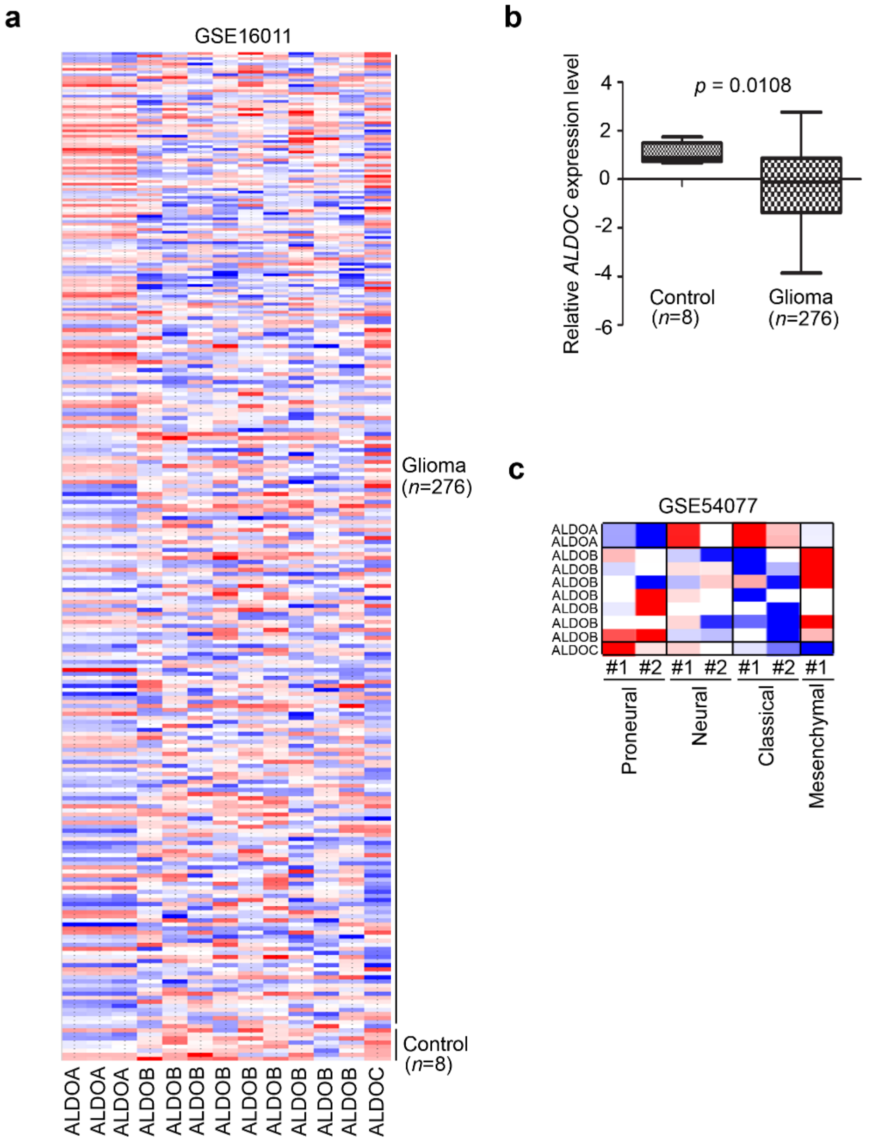

Figure S4. (**A**) A heat map showing the endogenous mRNA expression level of Aldolase family members *ALDOA*, *ALDOB*, and *ALDOC* in GSE16011 ($n = 284$). (**B**) A boxplot showing the distribution of *ALDOC* expression in clinical patients according to the normal tissues and tumor parts in the GSE16011 cohort ($p = 0.0108$). (**C**) A heat map showing the endogenous mRNA expression level of Aldolase family members *ALDOA*, *ALDOB*, and *ALDOC* in GSE54077 ($n = 7$). The significance of the differences in B was analyzed using a Student's *t*-test. All cut-off values were set before analysis, and all tests were two-tailed.

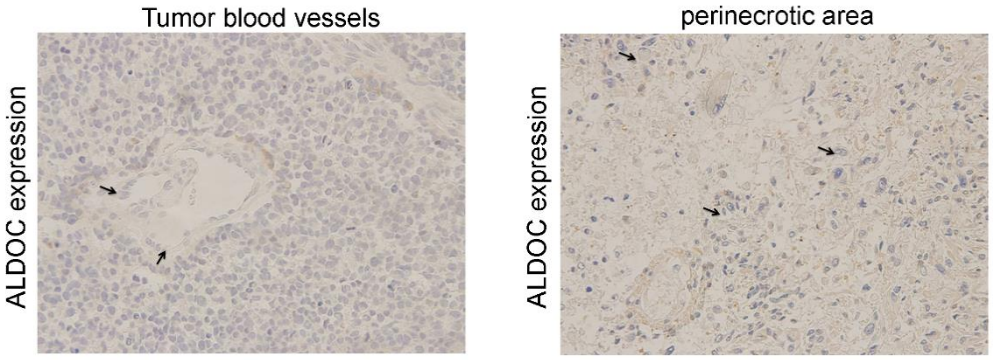

Figure S5. ALDOC protein level by immunohistochemistry in representative brain tumor tissues.

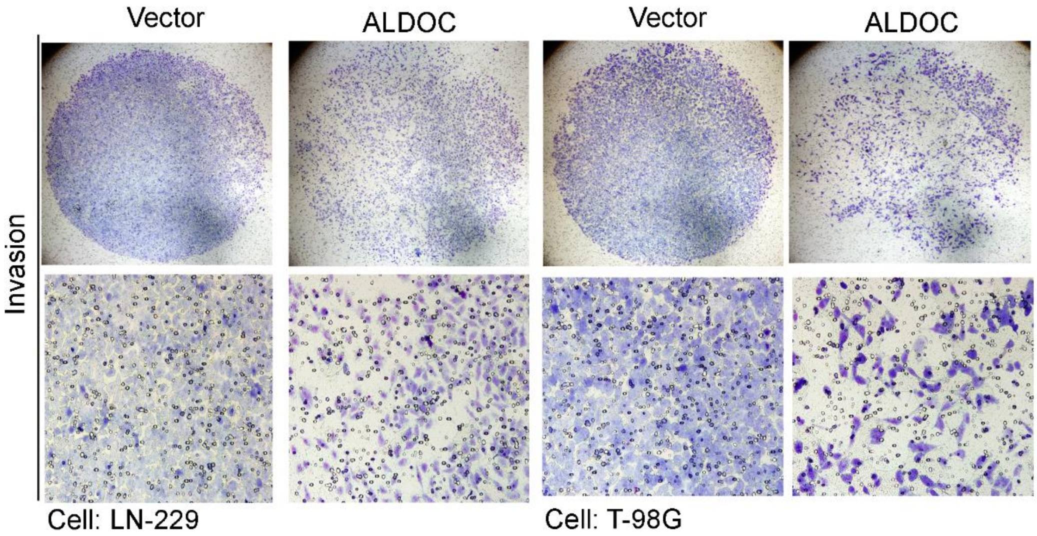

Figure S6. Representative Giemsa staining to estimate the invasion abilities of LN-229 and T-98G cells with forced expression of the vector control or exogenous ALDOC gene, respectively.
